# Case Report: Exome Sequencing Identified Variants in Three Candidate Genes From Two Families With Hearing Loss, Onychodystrophy, and Epilepsy

**DOI:** 10.3389/fgene.2021.728020

**Published:** 2021-11-29

**Authors:** Yuan Li, Jianjun Xiong, Yi Zhang, Lin Xu, Jianyun Liu, Tao Cai

**Affiliations:** ^1^ China-Japan Friendship Hospital, Beijing, China; ^2^ College of Basic Medical Science, Jiujiang University, Jiujiang, China; ^3^ Angen Gene Medicine Technology, Beijing, China; ^4^ Experimental Medicine Section, National Institute of Dental and Craniofacial Research (NIDCR), National Institutes of Health (NIH), Bethesda, MD, United States

**Keywords:** DDOD (dominant deafness-onychodystrophy), hearing loss, whole-exome sequencing (WES), ATP6V1B2, TJP2, KIF11

## Abstract

A cohort of 542 individuals in 166 families with congenital hearing loss was recruited for whole-exome sequencing analysis. Here, we report the identification of three variants in five affected individuals in two unrelated families. In family 1, a nonsense mutation (c.1516C>T, p.R506*) in the *ATP6V1B2* gene, a known causal allele for dominant deafness-onychodystrophy (DDOD), was identified in the mother and son with DDOD. However, a novel heterozygous variant (c.1590T>G, p.D530E) in *TJP2*, a known causal gene for hearing-loss, was also detected in the patients. In family 2, the same mutation (c.1516C>T, p.R506*) of *ATP6V1B2* was detected from the father and daughter with DDOD. Furthermore, a novel heterozygous variant (c.733A>G, p.M245V) in the *KIF11* gene was identified from the spouse with sensorineural hearing-loss and epilepsy. Notably, genotype-phenotype analysis of *KIF11*-associated disorders revealed that the p.M245V and two reported hearing-loss-associated variants (p.S235C and p.H244Y) are all mapped to a single β-sheet (Ser235∼M245) in the kinesin motor domain. Together, this is the first demonstration that *ATP6V1B2*-caused DDOD is an autosomal dominant genetic disease, compared to previous cases with *de novo* mutation. Our findings expand the variant spectrum of hearing-loss-associated genes and provide new insights on understanding of hearing-loss candidate genes *ATP6V1B2*, *TJP2*, and *KIF11*.

## Introduction

Hearing loss is one of the most common genetic defects in human beings (Morton and Nance, 2006), which is classified into syndromic hearing impairment (SHL) and non-syndromic hearing loss (NSHL). A total of 124 causative genes have been identified for NSHL (http://hereditaryhearingloss.org). Approximately 30% of affected individuals with hearing loss are considered to be syndromic (Tseng and Lalwani, 2000), whereas over 400 different syndromes involving hearing loss have been described (Gettelfinger and Dahl, 2018).

To investigate genetic causes of congenital hearing loss, a multi-institutional research collaboration recently started extensive medical examinations and next-generation sequencing for affected individuals. Among a cohort of 542 individuals in 166 families, four individuals in two families were affected with an extremely rare syndrome, mainly including hearing loss and onychodystrophy (DDOD). In addition, one family member in family 2 was affected with congenital hearing loss and epilepsy.

The cause of DDOD syndrome (MIM: 220500) was first identified, from three unrelated cases, to be a *de novo* heterozygous variant (c.1516C>T; p.R506*) in the *ATP6V1B2* gene (MIM: 606939) (Yuan et al., 2014). One additional single case with DDOD in different populations was also confirmed to be caused by the same variant (Menendez et al., 2017). The variant was also detected in a patient with broader clinical manifestation, including deafness, onychodystrophy, osteodystrophy-related abnormalities and intellectual disability (mental retardation), or seizures (so called DOORS syndrome) (Zadori et al., 2020). More recently, the same mutation was detected in nine individuals with either DDOD syndrome (two individuals) or DOORS syndrome (seven individuals) (Beauregard-Lacroix et al., 2021).

This is the only LoF allele identified in the *ATP6V1B2* gene thus far. All other seven variants are heterozygous missense alleles, linked to various conditions such as neurodevelopmental disorder, Cutis laxa, Zimmermann-Laband syndrome, epilepsy, intellectual disability, and mild gingival and nail abnormalities (HGMD). However, it remains undetermined whether this LoF variant (c.1516C>T; p.R506*) is also responsible for patients with family history segregating as an autosomal dominant (AD) form since the DDOD syndrome was first recognized in 1961 (Feinmesser and Zelig, 1961; Goodman et al., 1969; Moghadam and Statten, 1972; Kondoh et al., 1999; White and Fahey, 2011). Based on bioinformatics analysis on large populations like gnomAD database, it is possible that this LoF allele could not be passing on to the next generation because that the *ATP6V1B2* gene is an intolerant gene with high pLI value (Probability of being Loss of function Intolerant set at ≥0.9; pLI = 0.99 for *ATP6V1B2*) in gnomAD database (Lek et al., 2016).

In the present study, we show clinical findings of four individuals in two unrelated families affected with DDOD in AD form, and one individual affected with congenital sensorineural hearing loss and epilepsy in the family 2. By whole-exome sequencing (WES) analysis, we report the identification and characterization of three variants in three disease-associated genes (*ATP6V1B2*, *TJP2*, and *KIF11*) from five affected individuals in two families. Detailed genotype-phenotype analysis is applied for further characterizations of hearing loss-associated variants and potential pathogenesis.

## Materials and Methods

### Subjects

Five affected individuals in two unrelated families were recruited in the present study. Clinical tests were performed as routine for patients with hearing loss and additional conditions. Personal and familial medical history, including hearing loss, tinnitus, vestibular symptoms, use of aminoglycosides, and other clinical abnormalities, were collected. Written informed consents were obtained from participants or parents. This study and associated research protocols were approved by the Ethic Committee of participating Hospitals.

### Whole-Exome Sequencing and Bioinformatics Analysis

Genomic DNAs were extracted from peripheral blood cells. Whole-exome was captured by SureSelect Human All Exon Kit (Agilent), followed by high-throughput sequencing by HiSeq2000 sequencer (Illumina Inc.). After strict quality control, the clean reads were aligned to the human reference genome (hg19) for SNP calling and deleterious variants were predicted by multiple commonly used programs, such as MutationTaster, Polyphen-2, and SIFT, as described in previous study (Yu et al., 2016) . Detected variants with minor allele frequency (MAF) >0.001 in gnomAD or in-house Chinese Exome Database were excluded. ACMP/AMP classification of each of the variants were provided based on a standard criterion (Richards et al., 2015). Finally, potential pathogenic variants were verified by further Sanger sequencing with specific primers.

### Quantitative Real-Time PCR

To analyze a potential splicing variant, total RNAs were isolated from peripheral blood lymphocyte cells using TRIzol and reverse-transcribed using M-MLV reverse transcriptase (Invitrogen, Beijing section). RT-PCR was performed to analyze the c.1590T>G variant-containing region of *TJP2* using primers (forward: 5′-AGG​AAA​GGC​CAA​GTT​CCA​GA-3′ and reverse: 5′-GCA​TCC​TCC​CGC​ACT​AAT​CC-3′). The RT-PCR products were examined by 1.5% agarose gel electrophoresis and Sanger sequencing.

## Results

### Clinical Manifestations

**FIGURE 1:**
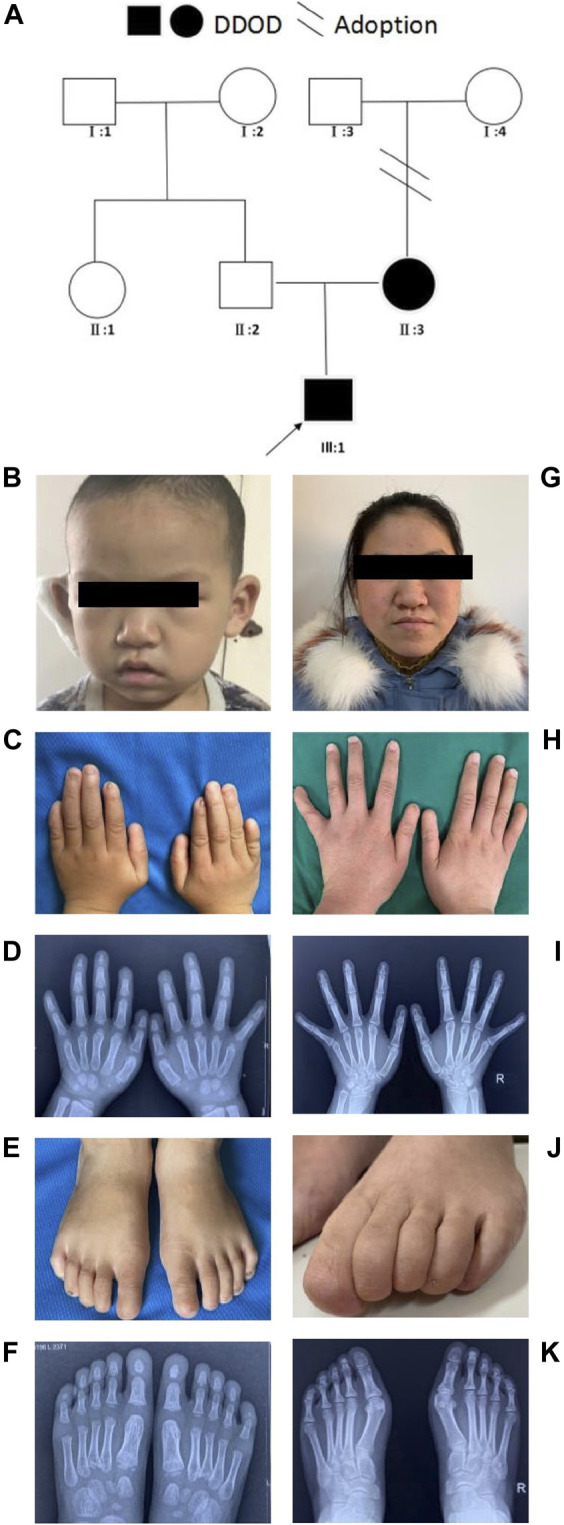
Clinical findings of family 1. **(A)** Family tree. **(B–F)** Proband and onychodystrophy. In fingers **(C,D)**, absence of first- and fifth-digits fingernails, index fingers aplasia, finger-like thumbs, and shorter fifth fingers. In toes **(E,F)**, absent toenails on the first and second digits, and hypoplasia toenails on the third to fifth digits of right foot; Left foot, absent toenails on the digits 1–3, rudimentary digits 4–5. Hand X-ray: distal phalanges absence of the index finger; hypoplasia of the distal phalanx of feet’s fifth digits. **(G–K)** Phenotypes of onychodystrophy of the mother are similar to the proband.

In family 1, the 6-year-old boy (Figures 1A,B) was first referred to the clinic when he was 20-month-old due to congenital hearing-loss. His hearing and speech performance became normal after receiving unilateral cochlear implantation at the age of 20 months. Physical examination showed bilateral nail anomalies in his fingers and toes. The fingernails of the first and fifth digits were absent, aplasia nails were exhibited on his index fingers, and the nails surfaces of the remaining fingers were rough and bumpy. The proband’s thumbs were fingerlike but not triphalangeal. His fifth fingers were abnormally short. X-ray analysis revealed bilateral absence of the distal phalanges of the index finger (Figures 1C,D). In his right foot, there were absent toenails on the first and second digits with severe hypoplasia toenails on the third to fifth digits. In the left foot, there were absent toenails on the digits 1–3, rudimentary and rough nails of the digits 4–5. His feet examination and roentgenograms showed bilateral hypoplasia of the distal phalanx of the fifth digits (Figures 1E,F). Audiological evaluations by behavioral audiometry, auditory brainstem response (ABR), multifrequency steady-state evoked potential (ASSR), and distorted product otoacoustic emission (DPOAE) revealed bilateral profound sensorineural hearing loss (SNHL) (Supplementary Figure S1). Temporal bone CT scan and brain MRI were normal. The 20-year-old mother (Figure 1G) with normal cognitive function also showed similar phenotypes, such as bilateral profound congenital sensorineural hearing loss and onychodystrophy (Figures 1H,I). Her toenail development was severely affected, showing no toenails at all (Figures 1J,K). There was no hearing intervention for the mother, and she communicates mainly through sign language.

**FIGURE 2:**
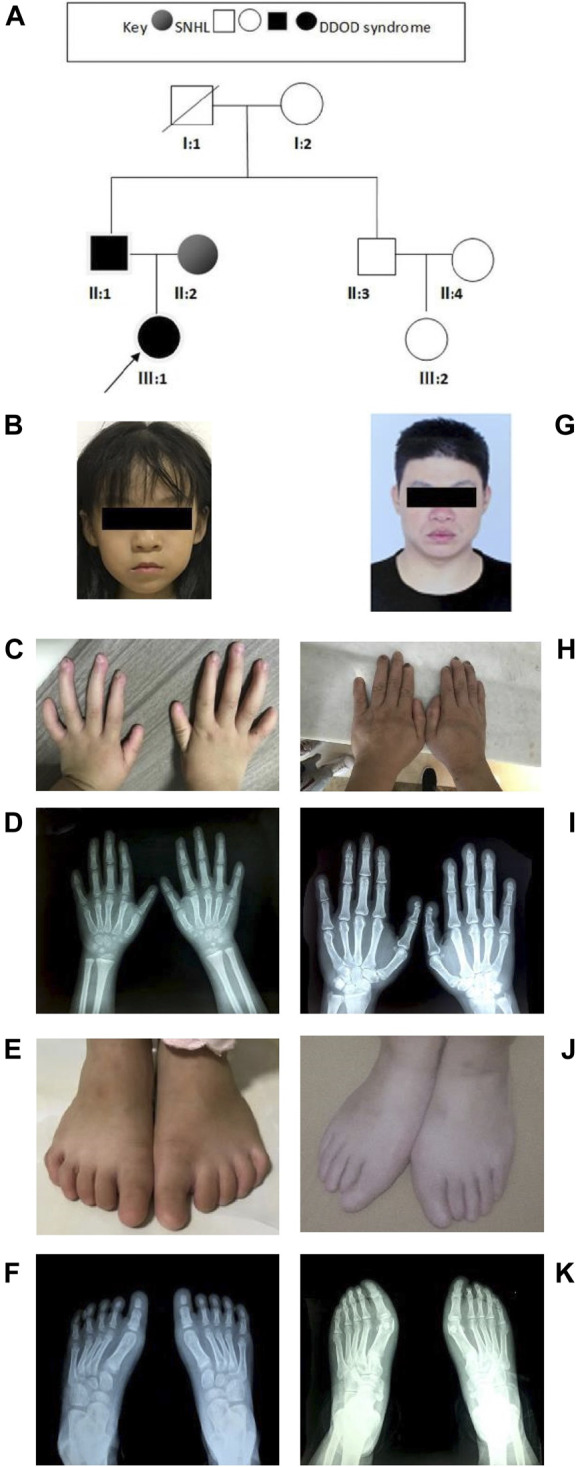
Clinical findings of family 2. **(A)** Family tree. **(B–F)** Proband and onychodystrophy. Absent fingernails on the first- and fifth-digits hypoplastic fingernails. Toenails are totally absent. Hand and foot X-rays show normal phalanx development. **(G–K)** Phenotypes of onychodystrophy of the father are similar to the proband.

In family 2 (Figure 2A), the 7-year-old girl (Figure 2B) did not pass the neonatal hearing screening. Audiological examinations by behavioral audiometry, ABR, ASSR, and DPOAE confirmed that she had bilateral profound sensorineural hearing loss. At 3 years of age, she received unilateral cochlear implantation. Her Categorical Auditory Performance (CAP) and Speech Intelligibility Rating (SIR) scores were 4 and 3 points, respectively. She was born vaginally at term (39W^+3^) after an uneventful pregnancy. There was no history of ototoxic drug use during pregnancy and no asphyxia and no history of injury at birth. Her growth measurements in terms of body weight, height, and head circumference were within normal ranges. She was not good at active expression and showed poor memory. Physical examination revealed her bilateral nail anomalies in fingers and toes (Figures 2C–F). Her fingernails in the first and fifth digits were absent, and the remaining fingers were apparently hypoplastic. Her thumbs were fingerlike but not triphalangeal; her left fifth digit was abnormally short. Her toenails were totally absent. X-ray showed normal phalanxes development. Her feet were flat (pes planus). Dental examinations showed her tooth hypoplasia. Physical development and neurological examinations by EEG, Griffith intelligence test, and behavioral evaluation were normal. Temporal bone CT scan and brain MRI did not show any abnormalities.

The 37-year-old father (Figure 2G) showed similar phenotypes as his daughter, including profound congenital SNHL and onychodystrophy (Figures 2H, I). In addition, his foot X-ray showed hypoplasia of the distal phalanx in his second right toe (Figures 2J,K). His cognitive function and other development processes were normal. The 28-year-old mother was diagnosed with profound congenital bilateral sensorineural hearing loss, infrequent epilepsy, and mild intellectual disability. Brain MRI did not show abnormalities. Both her parents communicate with sign language. Her grandparents were phenotypically normal.

### Identification of a Pathogenic Variant in *ATP6V1B2*

Trio-WES analysis identified a known pathogenic variant (c.1516C>T; p.R506*) in the *ATP6V1B2* gene (GenBank: NM_001693) from four affected individuals with hearing loss and onychodystrophy, i.e., the affected mother and son in family 1 and the affected father and daughter in family 2. This variant was previously reported as a *de novo* variant in multiple cases with deafness and onychodystrophy (Yuan et al., 2014) as well as multiple cases with DOORS syndrome (deafness, onychodystrophy, osteodystrophy, mental retardation, and seizures) (Beauregard-Lacroix et al., 2021). In the present two families, this LoF allele could be passed to the next generation consistent with the form of autosomal dominant disorder.

Based on ACMG/AMP classification (Richards et al., 2015), this variant can be classified to PVS1 (based on previous functional study) (Yuan et al., 2014), PP3 (predicted to be deleterious), PP5 (pathogenic in ClinVar and DVD), and PP1 (co-segregation in family members). This variant is therefore classified as pathogenic.

### A Variant of *TJP2* Also was Detected in the Patients in Family 1

**FIGURE 3:**
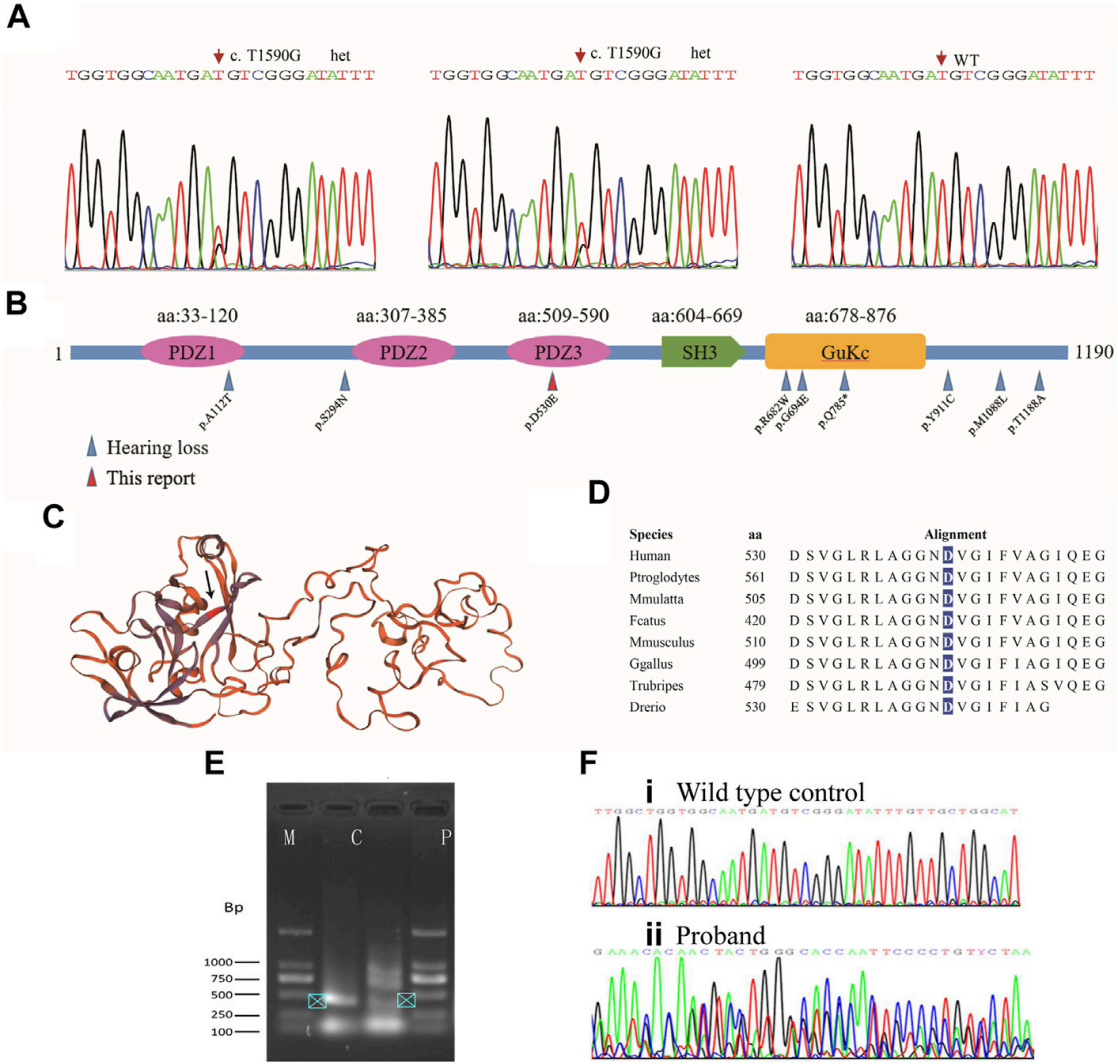
Mutation analysis of *TJP2*. **(A)** Sanger sequencing chromatograms show heterozygous variants in the affected son and mother. **(B)** All *TJP2* mutations curated in HGMD are mapped to the encoded TJP2 protein; Blue arrows: Variants detected in cases with hearing-loss; Red arrow: p.D530E in the PDZ3 motif detected in the study. Another variant (p.A112T) that caused hearing loss is mapped to PDZ1 motif. **(C)** Schematic of the TJP2 three-dimensional protein structure, the p.D530E position is indicated by an arrow. How the variants in PDZ motifs affect the protein structure are currently unknown. **(D)** The p.D530 residue is evolutionarily conserved from zebrafish to human. **(E)** Agarose gel electrophoresis shows PCR products of *TJP2* mRNA between c.1307 and 1712. M, marker; C, normal control sample; P, patient’s sample. **(F)** Sanger sequencing chromatograms show the TJP2 cDNA sequencing from normal control **(i)** and patient sample **(ii)**.

Further analysis of the trio-WES of the family 1 revealed that the affected mother and son also carried a variant (c.1590T>G, p.D530E; g.108844T>G; chr9:71845067T>G; Figure 3A) in the *TJP2* gene (GenBank: NM_004817), a known candidate gene for intrahepatic cholestasis and sensorineural hearing loss (Kim et al., 2014) (Supplementary Table S1). The c.1590T>G variant was predicted to be disease-causing (MAF = 0 in gnomAD) by MutationTaster and several commonly used algorithms. In addition, we identified a splicing site alteration in exon 9 in the *TJP2* gene (wildtype: CTGGTGGCAATGA**T**G; mutant: CTGGTGGCAATGA**G**G), thereby increasing a donor site at g.108836. To confirm the predicted splicing site, RT-PCR analysis was conducted to examine the c.1590T>G-containing region using the proband’s peripheral blood lymphocyte cells and normal control sample. The 406-bp RT-PCR product as a normal control was clearly detected by electrophoresis and further confirmed by Sanger sequencing (Figures 3E,F, arrowhead in lane C), while PCR product in patient was shown as multiple bands (lane P) and no single peak in Sanger sequencing, suggesting a mixed PCR product due to a splicing event. Quantification analysis using ImageJ showed at least 2-fold higher expression of 406-bp RT-PCR product (arrowhead) in control group (lane C) than that in patient (lane P), suggesting a potential effect of mutation on the accurate splicing of pre-mRNA into mRNA (Figure 3E). This variant in ACMG/AMP can be classified to PM2 (not shown in gnomAD or ChinaMap) and PP3 (predicted to be deleterious by multiple algorithms analyses). This variant is therefore classified as a VUS (variants of uncertain significance). Clinic spectrum comparison based on current medical data between these two patients and reported cases did not show significant differences. However, clinic follow-up is required to examine if these patients develop intrahepatic cholestasis or additional conditions.

### Novel Variant in *KIF11* Identified in the Affected Spouse in Family 2

**FIGURE 4:**
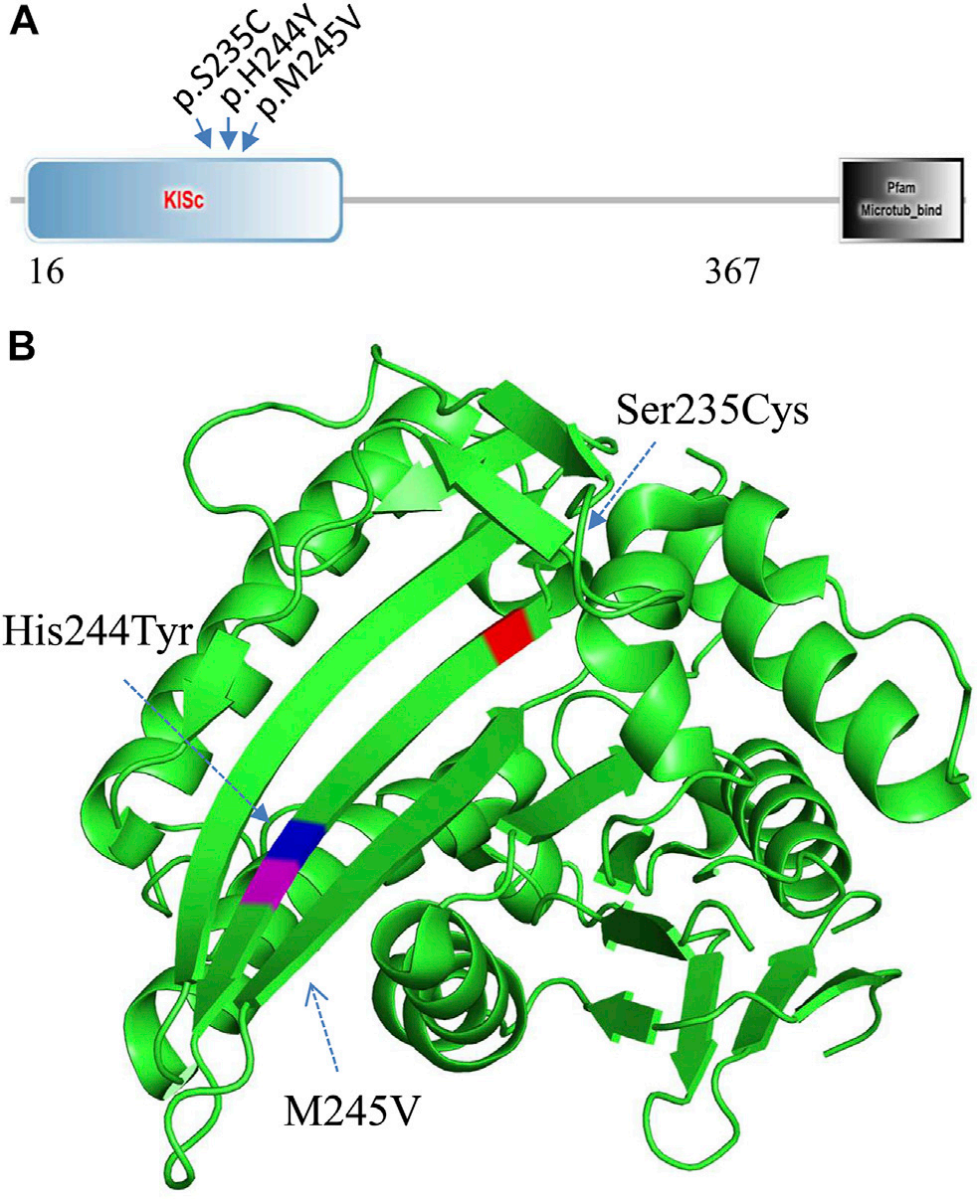
Mutation analysis of *KIF11*. **(A)** Three hearing-loss associated variants are located in the KISc domain (SmartMotif). **(B)** Based on the human KIF11 kinesin domain 3D-structure (PDB, 3K5E), all the three hearing-loss associated variants are located in the same beta-sheet in the Kinesin motor domain.

WES analysis of the affected spouse in family 2 (individual II-2, Figure 2A) identified a missense variant (c.733A>G, p.M245V in the *KIF11* gene) (GenBank acc. no., NM_004523). This variant is predicted to be disease-causing and is not recorded in gnomAD or ChES database (>10,000 Han population). At least 100 different mutations of the gene have been reported in patients with autosomal dominant microcephaly with or without chorioretinopathy, lymphedema, or mental retardation (MIM: 152950 and HGMD). Among them, two missense mutations (p.S235C and p.H244Y) in *KIF11* were previously identified in two unrelated patients with severe hearing loss in addition to chorioretinopathy and microcephaly (Jones et al., 2014; Mirzaa et al., 2014). Unexpectedly, we found that these two hearing-loss-associated variants and the current variant in *KIF11* (Figure 4A) are all mapped to a small region within a single β-sheet (amino acids 235–245, Figure 4B) in the catalytic domain of Kinesin motor (Smart Motif: mart.embl-heidelberg.de; PDB database: 3K5E), suggesting a specific portion of the protein implicated in hearing function. According to ACMG/AMP classification, this variant can be classified to PM2 (not detected in gnomAD and ChinaMap) and PP3 (predicted to be deleterious by multiple commonly used algorithms). This variant is therefore classified as a VUS (variants of uncertain significance).

## Discussion

*ATP6V1B2* encodes a component of vacuolar ATPase (V-ATPase), a multi-subunit enzyme that mediates acidification of intracellular organelles in endomembrane organelles, such as vacuoles, lysosomes, and endosomes (Lee et al., 1995). In embryonic mice, *ATP6V1B2* was expressed in the brain, inner ear, and nail (GenePaint), indicating its role in development of these tissues. To date, seven different variants in the *ATP6V1B2* gene are curated in HGMD. Only the LoF variant (c.1516C>T, p.[R506*]) at the very C-terminal region is repeatedly linked to hearing loss and onychodystrophy (Yuan et al., 2014), while other pathogenic variants are mainly associated with several other conditions as mentioned earlier. Consistent with a high pLI value of the gene in humans, *Atp6v1b2* knockout mice are embryonic lethal (MGI database, MP:0013292). Furthermore, *Atp6v1b2* c.1516C>T knockin mice displayed obvious cognitive defects and impaired hippocampal CA1 structure. However, hearing loss or abnormal cochlear morphology was not detected in the c.1516C>T knockin mice (Zhao et al., 2019), suggesting potential differences of hearing mechanisms between rodents and humans. Nevertheless, other possibilities such as gain-of-function effect and nonsense mediated mRNA decay (NMD effects) of the *c.1516C>T* in humans cannot be ruled out.

In this report, the LoF variant in *ATP6V1B2* is co-segregating with the four affected individuals in two unrelated families, which clearly demonstrates that the c.1516C>T allele is the genetic cause of DDOD in autosomal dominant form. Almost all previously reported cases with the c.1516C>T (Table 1) are caused by *de novo* mutation of this allele (Yuan et al., 2014; Menendez et al., 2017; Zadori et al., 2020; Beauregard-Lacroix et al., 2021). However, the underlying mechanism of how the c.1516C>T variant of *ATP6V1B2* causes DDOD or DOORS syndrome remains unknown. Apparently, patients with DOORS syndrome show broader defects involving brain and bone-related tissues.

**TABLE 1:** Comparison of patients with DDOD/DOORS due to c.1516C>T variant in *ATP6V1B2*.

	Yuan et al. (2014)	Menendez et al. (2017)	Zadori et al. (2020)	Beauregard-Lacroix et al. (2021)	This study
No. of patients	3	1	1	9^a^	4
Diagnosis	DDOD	DDOD	DOORS	DDOD/DOORS	DDOD
Inheritance	*de novo*	*de novo*	*de novo*	*Not defined* ^a^	AD
Mutation	Arg506^a^	Arg506^a^	Arg506^a^	Arg506^a^	Arg506^a^
Deafness	3/3	1/1	1/1	9/9	4/4
Onychodystrophy	3/3	1/1	1/1	9/9	4/4
Scoliosis/bone	NR	—	1/1	7/9	—
ID/Seizure	NR	—	1/1	7/9	—

—, absent; NR, not reported; AD, autosomal dominant; DDOD, dominant deafness–onychodystrophy syndrome; DOORS: deafness, onychodystrophy, osteodystrophy, and ID (intellectual disability) or seizures.

a Nine cases from eight unrelated families.

DDOD or DOORS syndrome can also be caused by additional candidate genes. For instance, dominant mutations in the *TBC1D24* gene (MIM: 613577) have been identified to cause hearing loss, while recessive mutations in the same gene are associated with DOORS syndrome (HGMD)(Campeau et al., 2014). In addition, deafness with nail dysplasia can be seen in Hay-Wells syndrome (MIM: 106260) and Fontaine progeroid syndrome (MIM: 612289). Therefore, further phenotypic, genetic, and functional studies are needed to explore how these syndromes are involved in deafness and onychodystrophy-associated pathways.

The *TJP2* gene encodes a protein of 1,190 amino acids, which is a member of the membrane-associated guanylate kinase homologous family (Itoh et al., 1999). In mouse embryos, *Tjp2* is highly expressed in liver, bile duct, and cochlear membranes connecting hair cells and supporting cells (Walsh et al., 2010), suggesting its role in liver and cochlear development. Functionally, *TJP2* is an integral part of the tight junction barrier between epithelial cells and endothelial cells and is essential for proper assembly of tight junctions (Traweger et al., 2003). In HGMD, 59 different variants in *TJP2* were linked to several conditions, such as intrahepatic cholestasis, hypercholanemia, and hearing loss. Ten hearing loss-associated variations are scattered in the encoded protein (Figure 3B; Supplementary Table S1). It is noted that only the c.2353C>T (p.Q785*) variant is linked to both cholestatic liver disease and hearing loss (Ehrenberg et al., 2019). In our case, the c.1590T>G (p.D530E) variation is located in the PDZ3 motif, which is predicted to implicate in interactions with scaffolding proteins.

*KIF11* (Kinesin Family Member 11) is a plus-end directed homotetrameric microtubule motor that functions during mitosis in microtubule crosslinking, antiparallel microtubule sliding, and bipolar spindle formation. In HGMA, 100 different variants in *KIF11* are linked to several conditions, such as microcephaly, lymphoedema, chorioretinopathy, and hearing loss. We found that the two reported hearing loss-associated variants (c.704C>G, p.[S235C] and c.730C>T, p.[H244Y]) and current variant (c.733A>G, p.[M245V]) in *KIF11* are clustered in the second beta-sheet in the catalytic domain of Kinesin motor. Presumably, this region might be involved in protein-protein interaction in CNS tissues including cochlear cells. Further studies are thus needed to clarify how *KIF11* is involved in the hearing process.

Taken together, we report here on five patients of two families with variants in three hearing loss associated genes. Our findings expand variant spectrums of hearing loss candidate genes, and shed new insights of pathogenetic effects of *ATP6V1B2*, *TJP2*, and *KIF11* on hearing functions.

## Data Availability

The original contributions presented in the study are publicly available. This data can be found here: https://www.lovd.nl/3.0/home. For ATP6V1B2: Variant ID: 0000813593. For TJP2: Variant ID: 0000813594. For KIF11: Variant ID: 0000813595.
