# Supplementary material for: Case Report: Exome Sequencing Identified Variants in Three Candidate Genes From Two Families With Hearing Loss, Onychodystrophy, and Epilepsy
Source: Front Genet. 2021 Nov 29;12:728020. doi: 10.3389/fgene.2021.728020 (PMC8667665; doi:10.3389/fgene.2021.728020)
Supplement: Supplementary file 1 [file Table1.docx]

**Suppl. Table 1**. Genotype-phenotype correlation of missense mutations associated with hearing loss in *TJP2*

| No. | Nucleotide  change | Protein  change | Domain | Reported phenotype | AD/AR | Ref. |
| --- | --- | --- | --- | --- | --- | --- |
| 1 | c.334G>A | p.A112T | PDZ1 | Hearing loss, non-syndromic | AD | (1) |
| 2 | c.881G>A | p.S294N | N/A | Sensorineural hearing loss | AD | (2) |
| New | c. 1590T>G | p.D530E | PDZ3 | Hearing loss with onychodystrophy | AD | This report |
| 3 | c.2044C>T | p.R682W | GuKc | Deafness | AD | (3) |
| 4 | c.2081G>A | p.G694E | GuKc | Hearing impairment, nonsyndromic | AD | (4) |
| 5 | c.2353C>T | p.Q785* | GuKc | Cholestatic liver disease and hearing loss | AR | (5) |
| 6 | c.2732A>G | p.Y911C | N/A | Hearing loss, progressive nonsyndromic | AR | (6) |
| 7 | c.3262A>T | p.M1088L | N/A | Deafness | AD | (3) |
| 8 | c.3562A>G | p.T1188A | N/A | Hearing loss, non-syndromic | AD | (1) |

Notes: A total of 8 different missense mutations associated with hearing loss in *TJP2* curated in HGMD database (Professional version) are listed here for a phenotypic comparison with the present case.

**References**

1. Kim MA, Kim YR, Sagong B, Cho HJ, Bae JW, Kim J, et al. Genetic analysis of genes related to tight junction function in the Korean population with non-syndromic hearing loss. PloS one. 2014;9(4):e95646.

2. Iwasa YI, Nishio SY, Usami SI. Comprehensive Genetic Analysis of Japanese Autosomal Dominant Sensorineural Hearing Loss Patients. PloS one. 2016;11(12):e0166781.

3. Zou S, Mei X, Yang W, Zhu R, Yang T, Hu H. Whole-exome sequencing identifies rare pathogenic and candidate variants in sporadic Chinese Han deaf patients. Clinical genetics. 2020;97(2):352-6.

4. Wang HY, Zhao YL, Liu Q, Yuan H, Gao Y, Lan L, et al. Identification of Two Disease-causing Genes TJP2 and GJB2 in a Chinese Family with Unconditional Autosomal Dominant Nonsyndromic Hereditary Hearing Impairment. Chinese medical journal. 2015;128(24):3345-51.

5. Ehrenberg M, Weiss S, Orenstein N, Goldenberg-Cohen N, Ben-Yosef T. The co-occurrence of rare non-ocular phenotypes in patients with inherited retinal degenerations. Mol Vis. 2019;25:691-702.

6. Gu X, Guo L, Ji H, Sun S, Chai R, Wang L, et al. Genetic testing for sporadic hearing loss using targeted massively parallel sequencing identifies 10 novel mutations. Clinical genetics. 2015;87(6):588-93.
