# Supplementary figures and images for: Case Report: Exome Sequencing Identified Variants in Three Candidate Genes From Two Families With Hearing Loss, Onychodystrophy, and Epilepsy
Source: Front Genet. 2021 Nov 29;12:728020. doi: 10.3389/fgene.2021.728020 (PMC8667665; doi:10.3389/fgene.2021.728020)

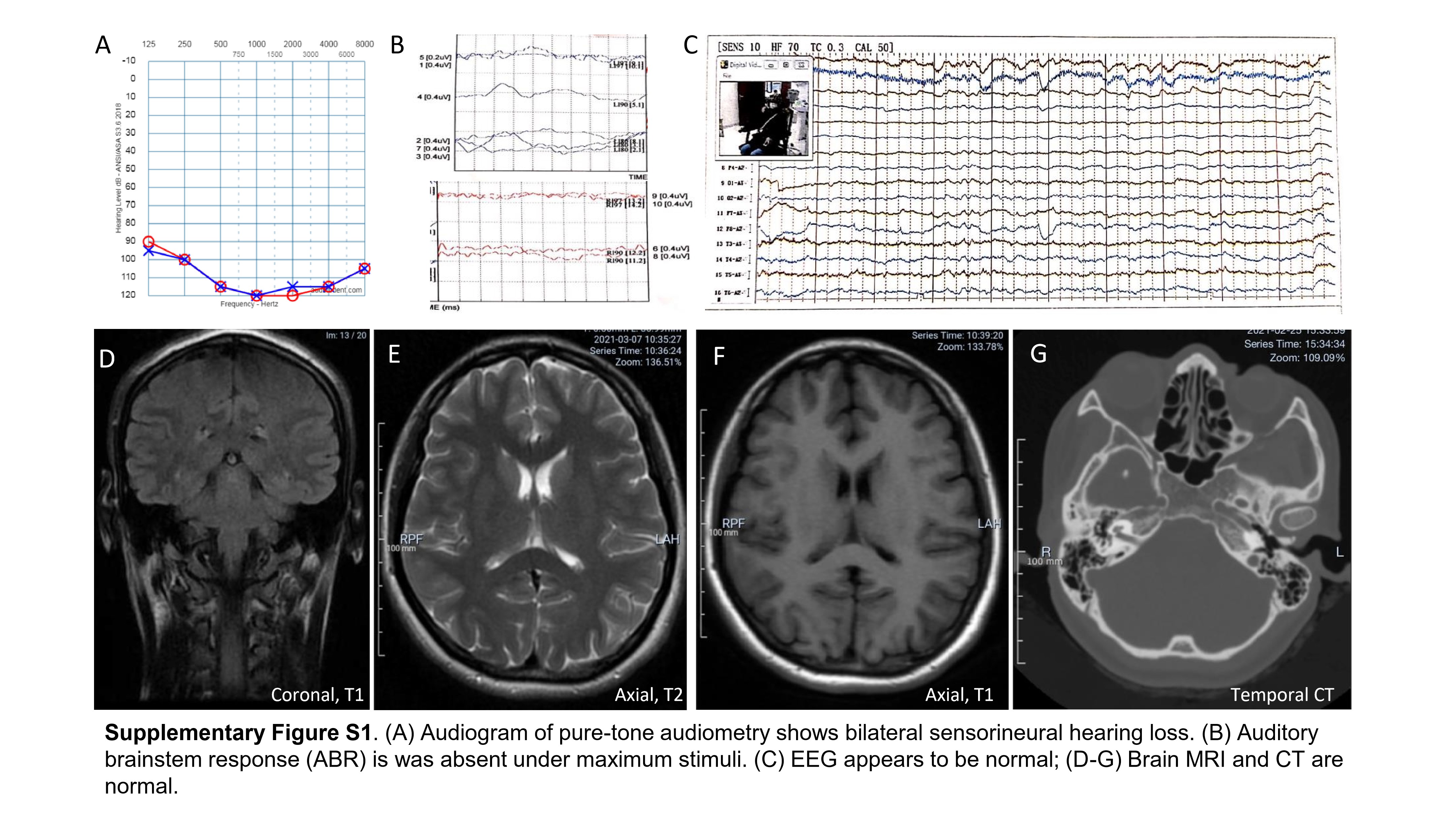

Supplement: Supplementary file 2 [file Image1.jpg]
